# Machine Learning Based Single-Frame Super-Resolution Processing for Lensless Blood Cell Counting

**DOI:** 10.3390/s16111836

**Published:** 2016-11-02

**Authors:** Xiwei Huang, Yu Jiang, Xu Liu, Hang Xu, Zhi Han, Hailong Rong, Haiping Yang, Mei Yan, Hao Yu

**Affiliations:** 1 Ministry of Education Key Lab of RF Circuits and Systems, Hangzhou Dianzi University, Hangzhou 310018, China; huangxiwei@hdu.edu.cn; 2 School of Microelectronics, Southeast University, Wuxi 214135, China; 220153639@seu.edu.cn (Z.H.); 220153658@seu.edu.cn (H.R.); 220153614@seu.edu.cn (H.Y.); 3 School of Electrical and Electronic Engineering, Nanyang Technological University, Singapore 639798, Singapore; yjiang017@e.ntu.edu.sg (Y.J.); liuxu16@bjut.edu.cn (X.L.); hxu011@e.ntu.edu.sg (H.X.)

**Keywords:** microfluidic cytometer, super-resolution, convolutional neural network, extreme learning machine, CMOS image sensor, point-of-care testing

## Abstract

A lensless blood cell counting system integrating microfluidic channel and a complementary metal oxide semiconductor (CMOS) image sensor is a promising technique to miniaturize the conventional optical lens based imaging system for point-of-care testing (POCT). However, such a system has limited resolution, making it imperative to improve resolution from the system-level using super-resolution (SR) processing. Yet, how to improve resolution towards better cell detection and recognition with low cost of processing resources and without degrading system throughput is still a challenge. In this article, two machine learning based single-frame SR processing types are proposed and compared for lensless blood cell counting, namely the Extreme Learning Machine based SR (ELMSR) and Convolutional Neural Network based SR (CNNSR). Moreover, lensless blood cell counting prototypes using commercial CMOS image sensors and custom designed backside-illuminated CMOS image sensors are demonstrated with ELMSR and CNNSR. When one captured low-resolution lensless cell image is input, an improved high-resolution cell image will be output. The experimental results show that the cell resolution is improved by 4×, and CNNSR has 9.5% improvement over the ELMSR on resolution enhancing performance. The cell counting results also match well with a commercial flow cytometer. Such ELMSR and CNNSR therefore have the potential for efficient resolution improvement in lensless blood cell counting systems towards POCT applications.

## 1. Introduction

Blood cell counts in point-of-care testing (POCT) provide critical information for rapid on-site disease diagnosis and monitoring [1,2]. For example, the counts of red blood cells (RBC, erythrocytes), white blood cell (WBC, leukocytes), and platelets help the diagnosis of anemia; the CD4+ lymphocyte count is used to monitor the progression of HIV/AIDS [3]. Existing techniques for blood cell counting mainly include manual counting using high magnification optical microscopy with high–numerical aperture objective lenses, or automated counting using commercial flow cytometers. However, manual counting is time-consuming, has low throughput, and the accuracy is easily affected by operators’ experiences, whereas commercial flow cytometers with bulky and sophisticated optics are prohibitively expensive. Hence, both are not suitable for POCT applications.

**Figure 1:**
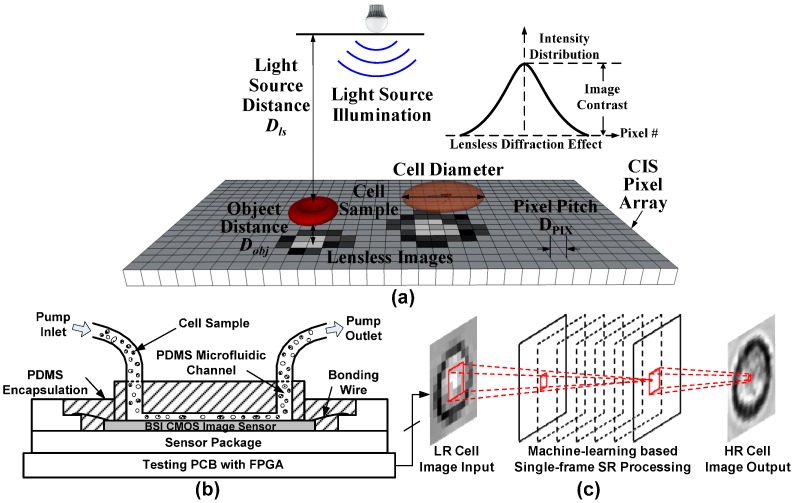
General lensless cell counting system setup based on CMOS image sensor (CIS). (**a**) lensless cell imaging principle; (**b**) cross-sectional view of the lensless system; and (**c**) concept of the machine-learning based single-frame super-resolution (SR) processing.

With the recent development of microfluidic lab-on-a-chip (LoC) technology and mass production of inexpensive CMOS image sensors (CIS), a miniaturized lensless imaging system becomes a competitive solution [4]. A lensless imaging system has a basic hardware setup, which directly integrates a microfluidic channel on a small CIS, and a white light source illuminates from above at a distance of *D_ls_* to sensor array [5]. When blood cell samples flow through the microfluidic channel at an objective distance *D_obj_* to the sensor array, their diffracted shadow images are recorded by the CIS underneath without any magnification by lens elements, as shown in Figure 1a. The spatial resolution of the diffracted image is mainly determined by the pixel pitch *D_PIX_* and affected by *D_ls_* and *D_obj_*. Shorter *D_obj_* leads to higher image contrast *C_im_*, hence less diffraction and better spatial resolution. This relation can be represented by the following expression:

(1) $$C_{im}=\alpha /\left(1+{\left(D_{obj}/D\right)}^{\varphi }\right),$$

where *α*, *D*, and φ represent three constants of contrast amplitude, characteristic distance, and shape parameter. Since both the cell size and *D_PIX_* are similar in scale (~µm), the captured cell shadow images are typically pixilated and suffer from low resolution, limiting the detection and recognition accuracy. Thus far, various lensless shadow imaging systems have been proposed for cell imaging, monitoring, and counting applications, etc., as summarized in Table 1. However, most of them suffer from low resolution for single cell imaging.

As analyzed in [6,7,8,9,10,11], the spatial resolution can be improved by increasing *D_ls_*, or decreasing *D_obj_* and *D_PIX_*. *D_ls_* can be as long as several centimeters, which is mainly determined by the size of the POCT system. *D_obj_* can be reduced by cutting off the protecting glass of the CIS and further removing the microlens and color filter layers on the sensor surface. *D_PIX_* is determined by the pixel fabrication process, and the state-of-the-art technology now reaches about 1.1–1.2 µm, equivalent to the size of platelets (~2 μm). However, pixel size cannot be further reduced as the amount of light incident on each pixel has decreased to a point that the signal-to-noise ratio and dynamic range loss would not compensate the resolution gain. Therefore, people are turning to other system-level solutions such as super-resolution (SR) processing.

SR processing is a technique that generates high-resolution (HR) images from low-resolution (LR) images [12]. Existing SR processing techniques applied in lensless imaging systems are mainly based on multi-frame reconstruction, in which multiple LR cell images with sub-pixel motions of the same object are synthesized into one single HR cell image. The sub-pixel motions can be generated by either flowing the cell samples through the microfluidic channel, shifting the light source, or sequentially activating multiple light sources at different locations [7,13,14]. However, the main problem for multi-frame SR is that the system needs to continuously capture, store, and process multiple LR images in order to recover one HR image, which not only limits the practical detection throughput but also requires large storage. Hence, it is not applicable for on-chip hardware implementation [7].

An alternative single-frame SR processing is thereby imperative [15]. Previous work introduces a computationally efficient single-frame SR approach by simply bilinear interpolating LR images [16]. Bilinear interpolation takes a weighted average of only the nearest 2 × 2 neighborhood pixel values based on the computed pixel’s distance from each of the known pixels. The required storage is only several pixels, hence it can be on-chip implemented for high processing speed. However, the recovered images are overly smooth, the sharpness of the edges cannot be maintained, and the high-frequency (HF) details cannot be recovered.

Recently, another category of machine learning based SR approaches is developing quickly [17,18,19,20,21,22,23,24,25]. Machine learning has very good performance and applications on a variety of problems such as visual/speech recognition, natural language processing, and biomedical imaging, etc. For example, in a POCT cell imaging system for waterborne pathogen detection, a machine learning algorithm has been adopted to automatically classify and distinguish *Giardia lamblia* cysts from other micro-objects based on the trained statistical features [17]. Also in cell biology, image-based screening relies on machine learning to efficiently recognize various phenotypes [18]. For SR processing, machine learning based approaches learn the correspondences between LR and HR image patches generated from a database of LR and HR image pairs, which are then applied to a new LR image to recover its most likely original HR image. The exemplary patches can be extracted either from external datasets [19,20], or the input image itself [21], or combined sources [22]. A pioneer work of [19] proposed an example-based learning strategy where the LR to HR prediction is learned via a Markov Random Field (MRF). Ref. [23] extends this work by using the Primal Sketch prior to enhance blurred edges, ridges, and corners. However, the above methods directly based on image patches typically require large databases of LR and HR patch pairs to incorporate any possible patterns encountered in testing, and are therefore computationally intensive. To reduce computational cost, [24] proposed a single image SR via sparse signal representation based on compressed sensing theory. Although the learned dictionary pair representation is more compact, its learning speed and optimization performances still need improvement.

In this paper, we tackle the aforementioned SR problems by employing two efficient machine-learning based approaches, namely Extreme Learning Machine (ELM) based SR and Convolutional Neural Network (CNN) based SR [26,27]. Similar to the widely used CNN in deep learning, ELM is also a general suite of machine-learning techniques. Both of them are lightweight, feed-forward, and possess the potential of on-chip hardware implementation. Based on ELMSR and CNNSR, prototypes of lensless blood cell imaging and counting are demonstrated using both commercial CIS and a custom designed back-side illuminated (BSI) CIS with smaller *D_PIX_* and *D_obj_*. Generic ELM and CNN based SR processing flows are as follows. Static HR cell images of different types are first off-line classified and stored as an HR image library to train an SR reference model. Next, with on-line input LR flowing cell images, single-frame SR processing is done using the reference model to reconstruct their corresponding HR images. Then, those cells can be accurately recognized and counted by only checking for the strongest structure similarity (SSIM) [28] referring to the off-line HR image library. Therefore, the developed microfluidic lensless cell counting system can achieve high single-cell image quality without throughput limitation, offering a cost-effective and portable solution for POC diagnostics.

## 2. Materials and Methods

### 2.1. Lensless Cell Counting System Design

#### 2.1.1. System Overview

The proposed lensless blood cell counting system is shown in Figure 1b, which is composed of one poly-dimethylsiloxane (PDMS) microfluidic channel bonded on the top surface of a CIS. The CIS chip can be either commercial or custom designed. During testing, an external syringe pump drives the cell sample solution through the channel continuously. Meanwhile, a white LED lamp illuminates the flowing cells from above. The cell shadow images are then continuously captured by the CIS underneath and output for processing by machine-learning based single-frame SR algorithms. The resolution of shadow images is improved such that recognition and counting of the flowing cells can be accurately performed afterward.

#### 2.1.2. CMOS Image Sensor

**Figure 2:**
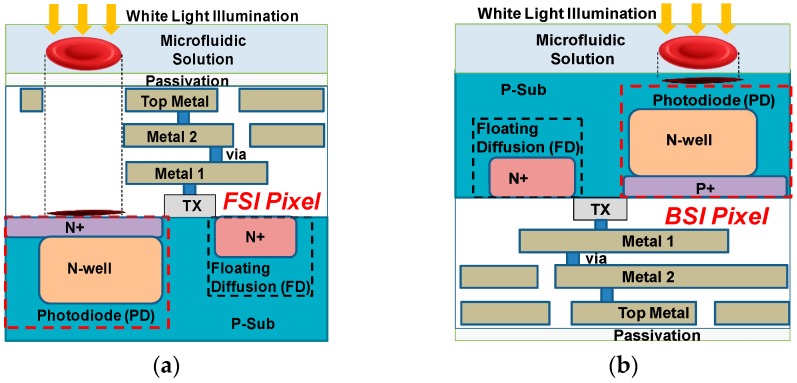
Comparison of front-side illuminated (FSI) and back-side illuminated (BSI) complementary metal oxide semiconductor (CMOS) image sensor pixel. (**a**) FSI pixel whose photodiode (PD) is far from the cell sample; and (**b**) BSI pixel whose PD is in close proximity with the cell sample.

To build the lensless blood cell counting system prototype, a commercial grayscale CMOS image sensor (MT9M032, Onsemi, San Jose, CA, USA) was first employed. Its pixel size is 2.2 μm × 2.2 μm, equivalent to normal platelet cells, and its array size is 1472 (H) × 1096 (V) with an active pixel area of 3.24 mm (H) × 2.41 mm (V). Before bonding with the microfluidic channel, the CIS protection glass and microlens layer were removed by a razor blade and plasma treatment (PDC-32G, Harrick Plasma, Ithaca, NY, USA), respectively. However, as the pixels of MT9M032 adopt a front-side illuminated (FSI) structure, shown in Figure 2a, it was necessary to coat a thin PDMS film on the sensor die before bonding with the microfluidic channel. There are two reasons for this. First, it would encapsulate and protect the sensor top circuit. Second, the sensor surface would be flatter so that the bonding can be tighter. The film was spin coated at a speed of 9000 rpm, and the minimum layer thickness realized was 6 μm. 

Nevertheless, an extra PDMS layer would increase the object distance *D_obj_* and degrade the contrast of lensless shadow image. FSI CIS also inherently suffers from low light sensitivity due to light degradation from stacking metal layers above photodiodes (PDs). Therefore, we specifically designed one back-side illuminated (BSI) CIS with an even smaller pixel pitch, 1.1 µm, and a 3.2-Mega pixel array. It has a die area of 5 mm × 5 mm and a photosensitive area of 1.7 mm × 2.2 mm. Different from FSI CIS, PD in BSI CIS is fabricated on the top layer without metal blocking the incident light, as shown in Figure 2b. Thus, the blood cell sample can directly contact the pixel surface with minimum distance. Higher sensitivity and quantum efficiency could also be achieved. Meanwhile, different from commercial FSI CIS, the top layer of the BSI sensor was a flat and smooth silicon and silicon dioxide layer without a microlens, which was suitable for direct microfluidic channel integration.

#### 2.1.3. Microfluidic Channel

In microfluidic channel design, to fully use the active pixel region that can capture flowing cells in the channel and prevent missing cells at high flow rates, the channel needs to fit in the sensor die on a diagonal so that its length is maximized. In the commercial CIS based prototype, the channel length was designed as 4.6 mm. In the BSI CIS based prototype, the channel length was 2.6 mm. A channel width of 500 μm was designed for both prototypes, as the relative wide channel is beneficial to reducing the clogging effect when cell samples of high concentration flow through. The microfluidic channel height was 30 μm such that the channel roof is just higher than the diameters of common blood cells and tumor cells. This ensured that the cells flow in close proximity to the sensor surface to generate higher contrast lensless images.

The microfluidic device was fabricated using the soft-lithography technique as presented previously [4]. Briefly, the microchannel mold was fabricated by patterning photoresist SU-8 (SU-8 25, Microchem, Westborough, MA, USA) on a silicon wafer. After that, PDMS (Sylgard 184, Dow Corning, Auburn, MI, USA) and curing agent were mixed at 10:1 volumetric ratio and cast onto the SU-8 mold. Then, the PDMS replica was peeled off from the mold after degassing and curing. The inlet and outlet were later punched to connect the microfluidic channel input with a syringe pump (KDS Legato180, KD Scientific Inc., Holliston, MA, USA), and channel output with a waste bin. To bond the PDMS microfluidic channel with the CIS chip, both surfaces were washed by ethanol first, and then cleaned with oxygen plasma for 25 s, and finally bonded together. After bonding, the gap between sensor die and package frame was filled with PDMS and then baked to encapsulate the bonding wires. The bonding strength is estimated to be about 30 kPa [29]. Note that, before each testing, we further coated the channel with 1% bovine serum albumin (BSA) in phosphate-buffered saline (PBS, Fisher Scientific, Pittsburgh, PA, USA) solution to improve wettability. After each test, the microfluidic channel should be washed using high flow rate distilled water so that it can be reused, just like the washing step in a commercial flow cytometer. 

#### 2.1.4. Testing Board

**Figure 3:**
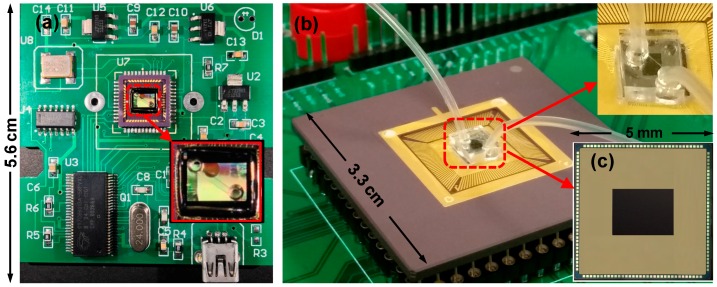
Testing board for lensless blood cell imaging. (**a**) Lensless system using commercial FSI CIS; (**b**) packaged BSI CIS integrated with the microfluidic channel and elastic thin tubing; and (**c**) custom designed BSI CIS chip.

The commercial CIS chip was soldered on one custom-designed 5.6 cm × 5.6 cm printed circuit board (PCB) that provided the sensor with power supplies and transferred data with external through a USB interface (CY7C68013-56 EZ-USB FX2, Cypress, San Jose, CA, USA), as shown in Figure 3a. An enlarged figure of PDMS microfluidic device on the chip is shown as an inset of Figure 3a. The BSI CIS chip was fabricated in a 65 nm BSI CMOS image sensor process and bonded to a 144-pin ceramic pin grid array (CPGA) package as shown in Figure 3b,c. The packaged BSI CIS chip was also mounted on one small field-programmable gate array (FPGA) testing board (XEM3010, Opal Kelly, Portland, OR, USA) to build a prototype system. The design details of the BSI CIS chip are out of the scope of this article so they are not introduced here. 

In testing, cell samples were continuously driven into the microfluidic channel at a typical flow rate of ~5 μL/min. The light source distance *D_ls_* was set as 12 cm. The light intensity at the sensor surface was set as 1.5k Lux. The sensor working status such as region-of-interest, exposure time, and the frame number to capture were set externally. The readout LR frames were buffered and processed to improve the resolution by ELMSR or CNNSR. Thus, the developed system could automatically recognize and count the flowing blood cells.

#### 2.1.5. Sample Preparation

Blood cell and HepG2 tumor cell sample solutions were prepared for testing. HepG2 cells (American Type Culture Collection, Baltimore, MD, USA) were cultured in Minimum Essential Media (MEM) supplemented with 10% fetal bovine serum, 1 mM sodium pyruvate, 0.1 mM MEM non-essential amino acids and grown in a T75 flask at 37 °C and a 5% CO_2_ atmosphere. The harvested cells were washed and re-suspended in PBS. The blood cell samples were collected from donators in Nanyang Technological University. Note that all volunteers signed written informed consent forms before enrollment, and all procedures comply with relevant laws and institutional guidelines, with the approval from the Ethics Committee of NTU on our research. To prevent cell aggregation, an ultrasonic cleaner (2510E-DTH, Branson Ultrasonics, Danbury, CT, USA) is applied to sonicate all of the samples for 10 min before input to the microchannel.

### 2.2. Machine-Learning Based Single-Frame SR Processing

After capturing the lensless images, digital image processing was performed for cell detection, resolution enhancement, cell type recognition, and cell counting of each type flowing through the microfluidic channel. The cell detection in each LR frame was realized by analyzing the temporal difference obtained by subtracting its previous background frame. After that, machine-learning based single-frame SR processing was performed. Next, mean structural similarity (MSSIM) index [28] was employed to characterize the similarity between the recovered HR image and the original HR images in the training library. The cell was categorized to the type that had the strongest MSSIM with HR training images. The final cell counting of each type was conducted by calculating the sum of increased cell numbers in all of the frames of flowing cells [4]. Here, the proposed two SR processing approaches, namely ELMSR and CNNSR, are elaborated and compared.

#### 2.2.1. ELMSR

**Figure 4:**
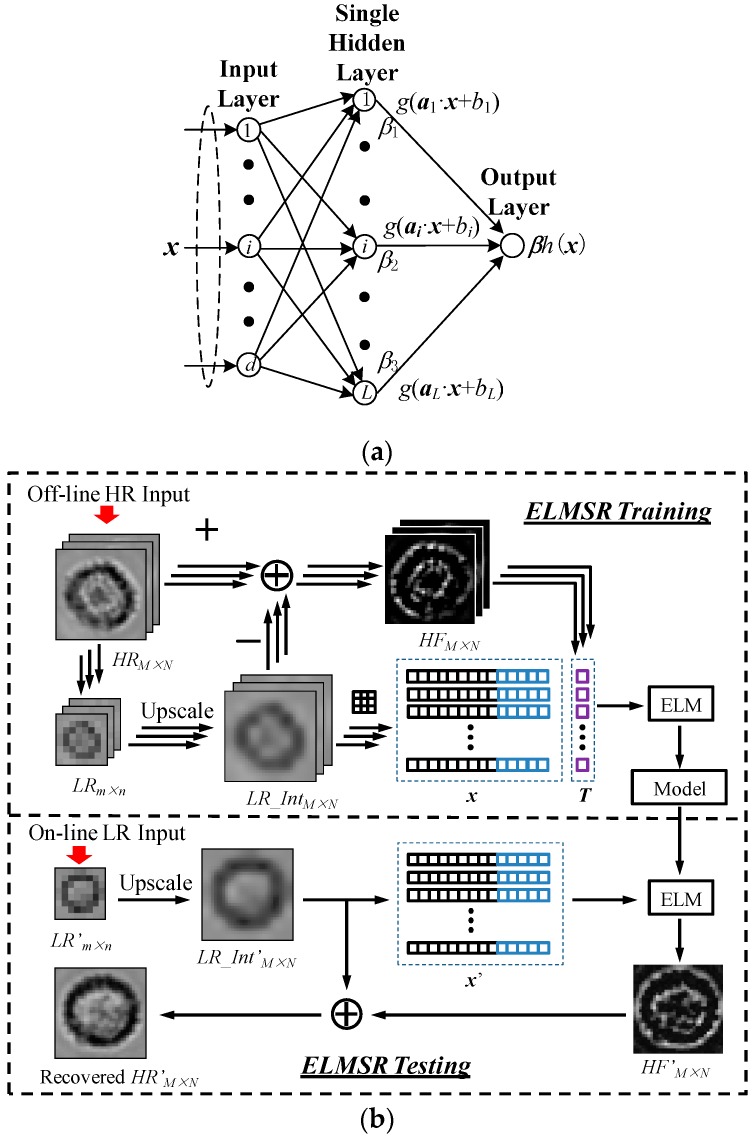
(**a**) Extreme learning machine (ELM) model structure; and (**b**) extreme learning machine based super-resolution (ELMSR) processing flow including one off-line training and one on-line testing step.

The ELM structure is a single-hidden-layer feed forward neural network [26], which has only one input layer, one single hidden layer, and one output layer as shown in Figure 4a. The ELMSR consists of two processing steps, namely off-line training and on-line testing steps. In the training step, a reference model is trained that can map the features in interpolated LR images with its HF components. These features include pixel intensity distribution, 1st order derivatives, and 2nd order derivatives, which represent the patterns of pixel intensity change. The HR training image library is constructed by capturing and storing the HR images of different cell types with various appearances using HR optical microscope camera.

The pseudo code for ELMSR is shown in Table 2. Firstly, *p* HR cell images are stored as the training library. For one HR image *HR_M×N_*, where *M* and N are the row and column pixel numbers, it is first bicubic down-sampled to one LR image *LR_m×n_*. Note that the down-sampled LR image is similar to the captured lensless LR image. The down sampling factor *t* determines the SR improvement factor, i.e., *M = m × t*, *N = n × t*. To obtain HF components, the LR image *LR_m×n_* is bicubically interpolated to *LR_Int_M×N_*, which is of the same size as *HR_M×N_* but blurred with HF details lost. Then, HF component *HF_M×N_* can be generated by subtracting the HR image *HR_M×N_* with the interpolated LR image *LR_Int_M×N_*,

(2) $$HF_{M\times N}=HR_{M\times N}-LR\_Int_{M\times N}.$$

After obtaining all *p* HF images *HF_M×N_*, their pixel intensity values will form a *p∙MN × 1* row vector as the training targeting value ***T***. Then, a 3 × 3 pixel patch *P*(*i*, *j*) is used to search through and extract the feature vector from *LR_Int_M×N_*, where *1 ≤ i ≤ M – 1* and *1 ≤ j ≤ N – 1*. Each patch creates a column vector consisting of nine pixel intensity values and ($\frac{\partial P}{\partial x},\frac{\partial P}{\partial y},\frac{\partial^{2}P}{\partial x},\frac{\partial^{2}P}{\partial y},\frac{\partial^{2}P}{\partial x\partial y}$), which indicates four 1st and 2nd order derivatives in the horizontal and vertical directions, as well as one 2nd order mixed derivatives. The column vectors extracted from all patches in *p* interpolated images *LR_Int_M×N_* form the feature matrix ***X***. Now, ***X*** and ***T*** form the ELM training dataset (***X***, ***T***). 

Next, after input the training dataset (***X***, ***T***) to ELM model, a row vector ***β*** containing the weights between all the hidden nodes and the output node are to be calculated. The ELM model has *d* input nodes, *L* hidden nodes, and one output node. The output of the *i*-th hidden node is

(3) $$h_{i}\left(x\right)=g\left(a_{i}\cdot x+b_{i}\right)=\frac{1}{1+\exp\left(-a_{i}\cdot x-b_{i}\right)},$$

where $a_{i}$ is a row vector of weights between all input nodes and the *i*-th hidden node; $b_{i}$ is a randomly generated bias term for the *i*-th hidden layer; g is a Sigmoid activation function of hidden layer. The output of ELM is

(4) f(x)=β·h(x),

where $h\left(x\right)={\left[h_{1}\left(x\right),h_{2}\left(x\right),\cdots ,h_{L}\left(x\right)\right]}^{T}$ is the output of the hidden layer. The output matrix of hidden layer is

(5) H(X)=G(AX+B),

where A is the weight matrix between input layer and hidden layer, B is the bias matrix, G is the same sigmoid function. Thus,

(6) T=βH(X).

In ELMSR, both training error and the norm of output weights should be minimized, i.e.,

(7) $$min\{\begin{array}{c} \left||\beta H\left(X\right)-T|\right|\\ \left||\beta |\right| \end{array}.$$

Thus, the orthogonal projection method can be applied to obtain β,

(8) $$\beta =T\cdot H{\left(X\right)}^{T}{\left[\frac{I}{C}+H\left(X\right)H{\left(X\right)}^{T}\right]}^{-1},$$

where C is a tuning parameter for the weight between ||βH(X)−T|| and ||β||, and I is the identity matrix with the same size to $H\left(X\right)H{\left(X\right)}^{T}$. The training data ***A***, ***B*** and ***β*** will be used as the ELMSR reference model.

In on-line testing, when a LR cell image *LR'_m×n_* is captured for processing, the corresponding HR image can be recovered using the same matrix ***A***, ***B*** and the trained weights ***β*** as follows. The *LR'_m×n_* is first bicubically interpolated by *t* times to *LR_Int'_M×N_*. The same patch searching used in ELMSR training is employed to extract the feature matrix X′ from *LR_Int'_M×N_*. Hence, the output vector can be obtained:

(9) $$f\left(X^{\prime }\right)=\beta H\left(X^{\prime }\right)=T\cdot H{\left(X\right)}^{T}{\left[\frac{I}{C}+H\left(X\right)H{\left(X\right)}^{T}\right]}^{-1}H\left(X^{\prime }\right),$$

Now $f\left(X^{\prime }\right)$ contains the recovered HF components $HF\prime_{M\times N}$. As such, the final HR image *HR'_M×N_* is recovered with sufficient HF details by

(10) $$HR\prime_{M\times N}=HF\prime_{M\times N}+LR\_Int\prime_{M\times N}.$$

As the resolution of lensless cell images is relatively low, we implemented a 4× magnification. Thus, a single cell LR shadow image of spatial size 12 × 12 can be improved to a 48 × 48 HR cell image. In the implemented ELM model, we set the node number in input, hidden, and output layer as *d* = 14, *L* = 20, and 1, respectively. Each 48 × 48 interpolated single cell image contains 46 × 46 = 2116 patches. The p training images will generate a feature matrix X of 2116*p* columns, and an HF intensity vector T with 2116p row. In testing, we set tuning parameter C = 512 to achieve a satisfied performance.

#### 2.2.2. CNNSR

**Figure 5:**
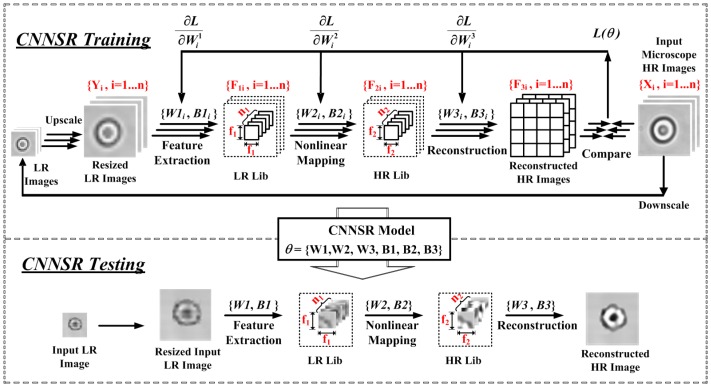
Convolutional neural network based super-resolution (CNNSR) processing flow including one off-line training and one on-line testing step.

As an alternate solution for optimized learning, CNNSR was proposed. Convolutional neural network (CNN) has been widely adopted in deep learning recently when dealing with large datasets of images. In CNNSR, the deep CNN can find a mapping function between LR and HR images. Similar to ELMSR, there is also one off-line training step for optimized model parameters that correlate the LR cell images with HR cell images, and one on-line testing step to improve the resolution of captured lensless image. The overall architecture of CNNSR is shown in Figure 5.

In CNNSR training, assume there are *n* training images, and the LR cell images in training library are first scaled up through bicubic interpolation to the same size as HR images. The interpolated images are denoted as *Y_i_*. The corresponding ground truth HR images are *X_i_*. The up-scaling factor is the SR magnification factor. An end-to-end mapping function *F* will be learned so that *F*(*Y_i_*) is as similar as possible to the original HR image *X_i_*. The mean squared error (MSE) between *Y_i_* and *X_i_* is applied as the loss function L(θ) to be minimized:

(11) $$L\left(\theta \right)=\frac{1}{n}\sum_{i=1}^{n}{\left||F\left(Y_{i};\theta \right)-X_{i}|\right|}^{2},$$

where *n* represents the number of training samples, and *θ* is the grouped network parameters of CNN that should be learned in the training step.

The pseudo code for CNNSR is shown in Table 3. The CNNSR mainly comprises three training layers as shown in Figure 5. The first layer randomly and densely extracts the overlapping patches from interpolated LR image *Y* and represents each patch as a high-dimensional vector:

(12) $$F_{1}\left(Y\right)=\max\left(0,W_{1}*Y+B_{1}\right),$$

where *W*_1_ represents *n*_1_ filters of spatial size *f*_1_ × *f*_1_ that convolute the input image *Y*; ‘*’ is the operation of convolution; *B*_1_ is an *n*_1_-dimensional vector indicating the biases, and each element of which is associated with a filter. The output vector *F*_1_(*Y*) consists of *n*_1_ feature maps. The rectified linear unit function ReLU(max(0, x)) is employed for the filter responses.

The second layer performs non-linear mapping of the *n*_1_-dimensional vectors to *n*_2_-dimensional ones, the operation is

(13) $$F_{2}\left(Y\right)=\max\left(0,W_{2}*F_{1}\left(Y\right)+B_{2}\right),$$

where *W*_2_ represents *n*_2_ filters of size *n*_1_ × *f*_2_ × *f*_2_, and *B*_2_ is an *n*_2_-dimensional bias vector. Hence, each output *n*_2_-dimensional vector is a representation of one HR patch that will reconstruct the final HR image.

The third layer performs final HR image reconstruction by aggregating the previous HR patches and generate one HR image that is as similar as possible to the original HR image *X*. Its operation is

(14) $$F_{3}\left(Y\right)=W_{3}*F_{2}\left(Y\right)+B_{3},$$

where *W*_3_ represents one set of filters of size *n*_2_ × *f*_3_ × *f*_3_, and *B*_3_ is a one-dimensional bias vector. The overlapping HR patches are averaged.

All the above three operations compose a CNN. The grouped network parameters *θ* = {*W*_1_*, W*_2_*, W*_3_*, B*_1_*, B*_2_*, B*_3_} shall be optimized together to get the mapping function *F* that minimizes the loss function *L*(*θ*)*.* This is achieved by stochastic gradient descent with the standard backpropagation. The weight matrices are updated as follows:

(15) $$\Delta_{i+1}=0.9\times \Delta_{i}+\eta \times \partial L/\partial W_{i}^{l},W_{i+1}^{l}=W_{i}^{l}+\Delta_{i+1}$$

where l∈{1,2,3}, *i* are the indices of layers and iterations, and η is the learning rate.

In an on-line testing step, when a new LR cell image *Y’* is captured by the lensless imaging system and input to CNNSR, the corresponding HR cell image *F{Y’}* can be recovered through the trained group network parameters *θ*. The input LR cell images are first extracted by *n*_1_ linear filters (*f*_1_ × *f*_1_). The extracted LR patches are then subtracted by its mean and projected into a dictionary with size *n*_1_. Later, a sparse coding solver is applied on the projected *n*_1_ coefficients to obtain *n*_2_ coefficients as the representation of HR patch. The sparse coding solver acts as a non-linear mapping operator that is fully feed-forward. After sparse coding, the *n*_2_ coefficients are projected into another HR dictionary for producing HR patches. Then, these overlapping patches are averaged and reconstructed to get final HR images.

In CNNSR, the magnification factor is also implemented as 4×. Due to the limited array size of single cells, the filter size *f*_1_ × *f*_1_ was set as 5 × 5, and *n*_1_ = 64. The *f*_2_ × *f*_2_ filter size was set as 1 × 1 with *n*_2_ = 32. In addition, the filter of the third layer set *f*_3_ = 3. Therefore, the calculation of a HR pixel adopts (5 + 3 − 1)^2^ = 49 LR pixel information, which leads to high restoration quality of CNNSR.

#### 2.2.3. Comparison of ELMSR and CNNSR

Both ELM and CNN are feed-forward neural networks. Thus, they are computing efficiently with little pre- or post-processing optimization. There is no need to resolve optimization problem on usage. A major merit of ELM is that the weights between the input layer and hidden layer are randomly generated, hence it is tuning-free without iterative training. Since the image number can be large if various cell types under different appearances are to be trained, ELMSR is suitable to speed up the training process. The advantage of using CNNSR is that the patch extraction and aggregation are directly formulated as convolutional layers. Hence, LR dictionary, HR dictionary, non-linear mapping and averaging are all involved in the filter optimization towards higher restoration quality. Note that the training of ELMSR and CNNSR model is done off-line. After the model is already trained, the computation would not need that much computation cost during testing. Moreover, ELMSR and CNNSR have the potential to be hardware implemented on-chip in the future. In that case, the computation would be much faster.

## 3. Results and Discussion

To evaluate the performance of ELMSR and CNNSR, both blood cell and tumor cell samples were tested. The resolution enhancement factor of 4× was selected. The Structural Similarity is employed as a metric to evaluate the quality of reconstructed images. 

### 3.1. Off-Line SR Training

**Figure 6:**
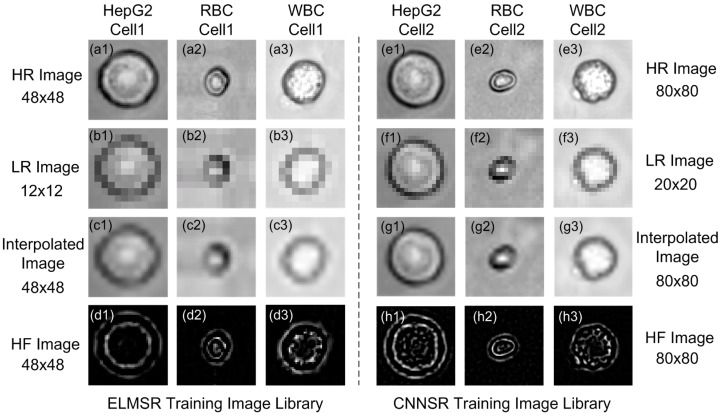
Example images of HepG2, Red blood cell (RBC), and white blood cell (WBC) in ELMSR and CNNSR training image libraries: (**a**) original high-resolution (HR) images with all high-frequency (HF) details in ELMSR library; (**b**) down-sampled low-resolution (LR) images with HF information lost in ELMSR library; (**c**) interpolated LR images whose HF cannot be recovered in ELMSR library; (**d**) HF components that are lost during downsampling in ELMSR library; (**e**) original HR images with all HF details in CNNSR library; (**f**) down-sampled LR images with HF information lost in CNNSR library; (**g**) interpolated LR images whose HF cannot be recovered in ELMSR library; and (**h**) HF components that are lost during down-sampling.

For both prototypes with ELMSR and CNNSR, the off-line training image libraries of blood cells and HepG2 tumor cells were first built. The HR training image of HepG2 and blood cells were captured using a microscope camera (Olympus IX71, Tokyo, Japan) and saved into the HR image library as shown in Figure 6a1–a3, e1–e3. Since there are two prototypes with different CMOS image sensors, the original HR images are saved as two different sizes, 48 × 48 and 80 × 80 corresponding to the ELMSR and CNNSR training image libraries. As the enhancement factor is four, we bicubic down-sampled the 48 × 48 HR cell images to 12 × 12 LR cell images, as shown in Figure 6b1–b3, and down-sampled the 80 × 80 HR cell images to 20 × 20 LR cell images, as shown in Figure 6f1–f3. Then, these LR cell images were interpolated back to 48 × 48 and 80 × 80, as shown in Figure 6c1–c3, g1–g3. Now, the detailed structures were already lost in the interpolated images as simple interpolation could not recover the HF components. Next, as shown in Figure 6d1–d3, g1–g3, the HF components for each training cell image were obtained by subtracting the original HR images with the interpolated cell images. Thus, the training library for ELMSR and CNNSR to train a reference model was generated. Different features in various cell types such as HepG2 tumor cell, RBC, and WBC could be clearly seen from the difference in their HF images. For the mixed HepG2 and blood samples, there are 30 HR images selected for each cell type to build the training library. Note that both interpolated images and HF images were used in ELMSR training to generate ELM reference models. But in CNNSR, interpolated images and HR images were directly employed to train the mapping function. We still keep Figure 6g1–g3 just to show the different HF features.

### 3.2. On-Line SR Testing

**Figure 7:**
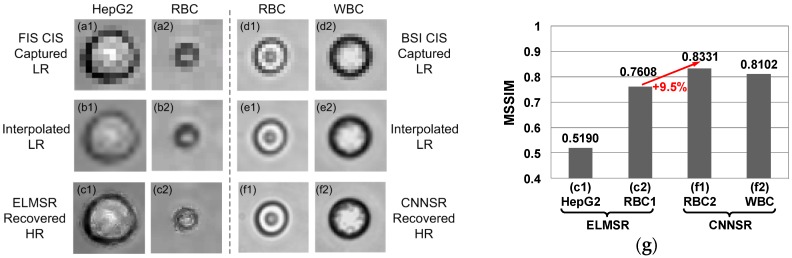
Example of HepG2, RBC, and WBC images in ELMSR and CNNSR testing: (**a**) raw LR images captured by FSI CIS with pixel pitch 2.2 μm; (**b**) interpolated LR images; (**c**) ELMSR recovered HR images; (**d**) raw LR images captured by BSI CIS with pixel pitch 1.1 μm; (**e**) interpolated LR images; (**f**) CNNSR recovered HR images, showing better performance in resolution improvement; and (**g**) the mean structural similarity (MSSIM) results for on-line recover cell images.

After building the off-line training image library and obtaining the training model, the on-line SR processing was performed when new lensless LR cell images were captured. As two CMOS image sensors of different pixel sizes (2.2 µm vs. 1.1 µm) were used to build the lensless imaging systems, the directly captured LR cell images were compared, as shown in Figure 7a,d. Due to the smaller pixel pitch of BSI CIS over the commercial FIS CIS, the captured LR RBC in Figure 7d1 was much clearer than Figure 7a2. The LR RBC images covered about 4 and 8 pixels at the diameter using FSI CIS and BSI CIS, respectively. These results demonstrated the advantage of using CIS of the smaller pixel pitch in generating LR lensless images of higher spatial resolution.

After the raw LR cell images were captured, the interpolated HR images could be generated, as shown in Figure 7b,e. The recovered HR images of one HepG2 cell and one RBC using the ELMSR model were shown in Figure 7c1,c2. The recovered HR images of one RBC and one WBC using the CNNSR model were shown in Figure 7f1,f2. Comparing the interpolated images in Figure 7b,e with SR recovered images in Figure 7c,f, it can be clearly observed that no matter which SR was used, the recovered images show more cell internal and edge information. Comparing the performance of resolution improvement for CNNSR and ELMSR, it can be seen that the HR images recovered by CNNSR have less noise compared with ELMSR. In Figure 7c2, the cell edge recovered by ELMSR still had some blur effect. However, in the CNNSR recovered HR images in Figure 7f1,f2, there was no such blur effect. Especially in the recovered WBC HR image in Figure 7f2, the cell membrane and nucleolus could be clearly seen. As shown in Figure 7g, the MSSIMs for HepG2 in Figure 7c1, RBC in Figure 7c2,f1, and WBC in Figure 7f2 with the corresponding HR image libraries are obtained as 0.5190, 0.7608, 0.8331, and 0.8102, respectively. Thus, CNNSR has 9.5% improvement over the ELMSR on resolution improvement quality. This is possibly due to fact that the filter optimization in CNNSR includes all the three CNN processing layers, while in ELMSR, there was no such joint optimization in training the network model. Note that although the input LR images for ELMSR and CNNSR are different due to the different CMOS image sensors used, the improved HR images are compared with their respective original HR images in their off-line training image libraries. Thus, the performance of SR improvement is directly evaluated by comparing the MSSIM metric.

### 3.3. On-Line Cell Recognition and Counting

The on-line cell recognition and counting performances of the developed prototype were further evaluated using mixed tumor cells and RBC samples. The RBC/HepG2 cell sample was prepared and measured by the commercial flow cytometer (Accuri C6, BD Biosciences, San Jose, CA, USA). The absolute counts of RBC and HepG2 are 1054 and 978, the ratio of which was about 1.08:1 (51.9%:48.1%). The sample was tested at a flow rate of 5 μL/min using the developed lensless system for six groups, with each group for one minute. The cell counts were obtained as shown in Table 4. The mean RBC/HepG2 ratio is 52.60%:47.40% = 1.11:1, and the coefficient of variation (CV) is 0.10, which matched well with the commercial flow cytometer result (1.08:1). Based on the current sample concentration, the average throughput was 3080 min^−1^. Although the throughput was relatively low compared with commercial flow cytometry, it can be further improved by increasing the sample concentration and flow rate, as the cells captured in each image and the total cells captured in a certain number of images are increased.

## 4. Conclusions

To tackle the low-resolution limitation in lensless microfluidic imaging towards POCT blood cell counting, ELMSR and CNNSR processing are proposed. Lensless blood cell counting prototypes integrating microfluidic channels with custom-designed back-side illuminated CIS and commercial front-side illuminated CIS were also developed. The experimental results demonstrated that the cell resolution could be improved by 4×, and CNNSR showed 9.5% improved quality over the ELMSR on resolution enhancing. The cell counting results also matched well with those of the commercial flow cytometer. 

Different from existing cell counting techniques without imaging information such as coulter counter, our imaging based methods can provide clear cell images that are intrinsically interesting to diagnostic users for single cell level analysis. As the imaging device in our system is a CMOS image sensor chip that can be mass produced, the cost is much lower compared with techniques based on lenses. Thus, it is also affordable for one-time usage to prevent cross contamination. Meanwhile, the computation efficient machine-learning SR processing has the potential to be directly hardware-implemented in the CMOS image sensor chip. Therefore, although the existing processing part is realized using software on a laptop, it has the potential to be all integrated on-chip to realize a much faster, really portable, automated, and cost-effective system. The developed lensless systems with machine-learning based single-frame SR processing are thus promising for future POCT applications.

## Figures and Tables

**Table 1:** Comparison of lensless shadow imaging systems.

Ref.	Description	Advantage	Disadvantage
[6]	LUCAS, static cell counting based on one single captured low-resolution (LR) image of a droplet of cell solution in between two cover glasses on CIS surface	Simple architecture and large field for cell counting	Low resolution single cell image
[7]	SROFM, drop and capillary flow cells through microchannel, capture multiple LR image to generate one high-resolution (HR) image	High resolution single cell image	Low throughput for cell counting
[8]	Static cell counting by dropping cell sample in a chamber over CMOS image sensor (CIS)	Multi-color imaging	Low resolution single cell image
[9]	Continuously monitor cells in incubator above CIS	Non-label continuous imaging	Low resolution single cell image

**Table 2:** Pseudo code for extreme learning machine based super-resolution (ELMSR).

ELMSR Training:
1 Downscale the input p $HR_{M\times N}$ to obtain p $LR_{m\times n}$ images
2 Upscale p $LR_{m\times n}$ images to p $LR_{Int_{M\times N}}$
3 Generate feature matrix ***X*** from p $LR_{Int_{M\times N}}$
4 Generate *p* $HF_{M\times N}$ and row vector ***T***
5 Generate the weight vector β with [***X***, ***T***]
T=βH(X)=βG(AX+B), $\beta =T\cdot H{\left(X\right)}^{T}{\left[I/C+H\left(X\right)H{\left(X\right)}^{T}\right]}^{-1}$
**ELMSR Testing:**
6 Input LR image $LR\prime_{m\times n}$ for testing
7 Upscale $LR\prime_{m\times n}$ to $LR\_Int\prime_{M\times N}$
8 Generate feature matrix ***X'*** from $LR\_Int\prime_{M\times N}$
9 Calculate $HF\prime_{M\times N}$ image, $T^{\prime }=\beta H\left(X^{\prime }\right)=T\cdot H{\left(X\right)}^{T}{\left[I/C+H\left(X\right)H{\left(X\right)}^{T}\right]}^{-1}H\left(X^{\prime }\right)$
10 Generate final SR output with HF components $HR\prime_{M\times N}=LR\_Int\prime_{M\times N}+HF\prime_{M\times N}$

$HR_{M\times N}$: original high-resolution cell image of size M × N. $LR_{m\times n}$: low-resolution cell image of size m × n. $LR_{Int_{M\times N}}$: interpolated low-resolution cell image of size M × N. $HF_{M\times N}$: high-frequency component of cell image of size M × N.

**Table 3:** Pseudo code for convolutional neural network based super-resolution (CNNSR).

CNNSR Training
**Input: LR cell images *{Y_i_}* and corresponding HR cell images *{X_i_}***
**Output: Model parameter** $\theta =\left\{W_{1},W_{2},W_{3},B_{1},B_{2},B_{3}\right\}$
1 θ are initialized by drawing randomly from Gaussian Distribution (μ=0,σ=0.001)
2 **For** i=0 to n // n is the number of training image
3 **For** l = 1 to 3 // 3 layers to tune
4 Calculate $F_{i}\left(Y\right)$ based on Equations (13)–(15)
5 **End For**
6 **Calculate** $L\left(\theta \right)=\frac{1}{n}\sum_{i=1}^{n}{\left||F\left(Y_{i};\theta \right)-X_{i}|\right|}^{2}$
7 **If** L(θ)<ε // ε is closed to zero
8 Calculate $\Delta_{i+1}=0.9\times \Delta_{i}+\eta \times \partial L/\partial W_{i}^{l}$, $W_{i+1}^{l}=W_{i}^{l}+\Delta_{i+1}$
9 **End If**
10 **End For**

**CNNSR Testing**
**Input: LR cell image *{Y’}* and Model parameter** $\theta =\left\{W_{1},W_{2},W_{3},B_{1},B_{2},B_{3}\right\}$
**Output: Corresponding HR cell images *F{Y’}***
11. **For** l = 1 to 3 // 3-layer network
12 Calculate F(Y′) based on Equations (13)–(15)
13 **End For**

**Table 4:** Measured counting results of mixed Red blood cell (RBC) and HepG2 sample.

Group	RBC (# μL^−1^)	HepG2 (# μL^−1^)	RBC/HepG2
1	239 (54.32%)	201 (45.68%)	1.19
2	338 (50.22%)	335 (49.78%)	1.01
3	260 (53.72%)	224 (46.28%)	1.06
4	435 (52.98%)	386 (47.02%)	1.12
5	340 (55.74%)	270 (44.26%)	1.26
6	334 (49.85%)	336 (50.15%)	0.99
Mean	324 (52.60%)	292 (47.40%)	1.11
Stdev	70	72	0.11
CV	0.22	0.25	0.10

CV: coefficient of variation.
